# Use of Nickel Oxide Catalysts (Bunsenites) for In-Situ Hydrothermal Upgrading Process of Heavy Oil

**DOI:** 10.3390/nano13081351

**Published:** 2023-04-12

**Authors:** Jiménez Padilla Pedro Alonso, Richard Djimasbe, Rustem Zairov, Chengdong Yuan, Ameen A. Al-Muntaser, Alexey Stepanov, Guliya Nizameeva, Alexey Dovzhenko, Muneer A. Suwaid, Mikhail A. Varfolomeev, Almaz L. Zinnatullin

**Affiliations:** 1 Department of Petroleum Engineering, Kazan Federal University, 420008 Kazan, Russia; 2 Department of Physical Chemistry, Kazan Federal University, 420008 Kazan, Russia; 3 Arbuzov Institute of Organic and Physical Chemistry, FRC Kazan Scientific Center, Russian Academy of Sciences, 420088 Kazan, Russia; 4 Department of Physics, Kazan National Research Technological University, 420015 Kazan, Russia; 5 Institute of Physics, Kazan Federal University, 420008 Kazan, Russia; almaz.zinnatullin@gmail.com

**Keywords:** synthesis, nickel oxides, catalyst, adsorption, heavy oil, viscosity, hydrothermal upgrading

## Abstract

In this study, Nickel oxide-based catalysts (Ni_x_O_x_) were synthesized and used for the in-situ upgrading process of heavy crude oil (viscosity 2157 mPa·s, and API gravity of 14.1° at 25 °C) in aquathermolysis conditions for viscosity reduction and heavy oil recovery. All characterizations of the obtained nanoparticles catalysts (Ni_x_O_x_) were performed through Scanning Electron Microscopy (SEM), Transmission Electron Microscopy (TEM), Atomic Force Microscopy (AFM), X-Ray and Diffraction (XRD), and ASAP 2400 analyzer from Micromeritics (USA), methods. Experiments of catalytic and non-catalytic upgrading processes were carried out in a discontinuous reactor at a temperature of 300 °C and 72 bars for 24 h and 2% of catalyst ratio to the total weight of heavy crude oil. XRD analysis revealed that the use of nanoparticles of NiO significantly participated in the upgrading processes (by desulfurization) where different activated form catalysts were observed, such as α-NiS, β-NiS, Ni_3_S_4_, Ni_9_S_8_, and NiO. The results of viscosity analysis, elemental analysis, and ^13^C NMR analysis revealed that the viscosity of heavy crude oil decreased from 2157 to 800 mPa·s, heteroatoms removal from heavy oil ranged from S—4.28% to 3.32% and N—0.40% to 0.37%, and total content of fractions (ΣC_8_–C_25_) increased from 59.56% to a maximum of 72.21%, with catalyst-3 thank to isomerization of normal and cyclo-alkanes and dealkylation of lateral chains of aromatics structures, respectively. Moreover, the obtained nanoparticles showed good selectivity, promoting in-situ hydrogenation-dehydrogenation reactions, and hydrogen redistribution over carbons (H/C) is improved, ranging from 1.48 to a maximum of 1.77 in sample catalyst-3. On the other hand, the use of nanoparticle catalysts have also impacted the hydrogen production, where the H_2_/CO provided from the water gas shift reaction has increased. Nickel oxide catalysts have the potential for in-situ hydrothermal upgrading of heavy crude oil because of their great potential to catalyze the aquathermolysis reactions in the presence of steam.

## 1. Introduction

Given the ever-increasing demand for and consumption of fuels around world, the exploitation, production, and processing of available hydrocarbon sources (heavy oil and natural bitumen) will bridge the gap of lack of supply. Consequently, their exploitation is not an easy task to achieve, because of high viscosity and low API gravity (high content of resins and asphaltenes, including heavy metals) [1,2]. A decrease of viscosity and density, as well as heteroatom and metal removal, are the main purpose of the hydrothermal upgrading process of heavy crude oil. Nowadays, many thermal methods, namely in-situ combustion (ISC), microwave heating (MWH), and dry pyrolysis, are applied for heavy oil recovery, despite the need for heating systems to heat the reservoir formation to reduce the viscosity of the oil, which often happens with a significant release of gases and coke formation, decreasing the yield of oil recovery [3,4,5,6,7,8]. The in-situ steam injection process has been used for decades to improve the rate of heavy oil recovery through three methods: steam-flooding, Cyclic Steam Stimulation (CSS), and, recently, Steam-Assisted Gravity Drainage (SAGD); of these, steam injection-based methods are the most widely applied [9]. However, several investigations have been conducted to highlight and develop thermal techniques to achieve the goal of heavy oil recovery. Thermal methods, including steam injection, in-situ combustion, hot water injection, etc., are the most well-known techniques and are usually applied to the production and processing of heavy oils by reducing the viscosity and increasing the flow characteristics [6,10,11]. As for in-situ combustion, although it has a high displacement capacity, its wide application is still limited due to its complexity, as well as the difficulty of controlling and predicting the burning front (e.g., combustion is unstable) [12]. Other methods, such as ultrasound and magnetic physical treatments, have also been advanced but have not yet been fully commercialized [13,14]. Steam injection is by far the most widely used thermal method for heavy oil recovery. Nevertheless, in the process of its application, many problems have been discovered; for example, for every 2–5 barrels of water injected as steam, one barrel of oil is produced, giving it significant energy consumption with a high environmental impact [15]. This implies that significant volumes of water and natural gas are needed for steam generation, which adds to the capital and operating cost. In addition, oil produced from steam-enhanced recovery processes requires the addition of expensive diluents to support pipeline transportation to refineries, as well as additional surface upgrading to meet refinery feedstock specifications. Therefore, any effort that helps to improve steam injection efficiency by solving or relieving one or more of the aforementioned problems is worth encouraging. Due to the search for new technologies for the improved recovery of heavy crudes, these processes have been studied and applied both conventionally and in a hybrid way in conjunction with solvents and catalysts. The application of catalysts during steam injection is intended to decrease the activation energy necessary for the production of hydrocarbons of lower molecular weight during the characteristic times of the process, thus achieving an in-situ improvement of the hydrocarbons. This allows an additional decrease in viscosity that could lead to a greater increase in the recovery factor. Studies have been reported on different metal-based catalysts (Fe, Co, Ni) [16,17,18]; however, it has been shown that Ni is the most active catalyst for the hydrocracking and hydrogenation of heavy components, while inhibiting condensation and recombination reactions, as demonstrated by Muneer et al. in their study [2]. Several studies of hydrothermal upgrading of heavy oil using different catalysts have been performed [19,20,21,22,23,24,25,26,27,28,29,30,31,32]. Hamedi-Shokrlu et al. [19] studied the effect of nickel nanoparticles on the in-situ upgrading of heavy oil in aquathermolysis conditions. Based on the kinetic analysis, they concluded that nickel nanoparticles reduced the activation energy of the reactions corresponding to the hydrogen sulfide generation by approximately 50%. Hart et al. [20] studied the in-situ catalytic (bimetallic) upgrading processes of heavy oil using a pelletized Ni-Mo/Al_2_O_3_ catalyst in the THAI process. The results revealed that the cleavage of the C-C, C-S, C-N, and C-C bonds is facilitated by the acid sites of the alumina support of the Ni-Mo/Al_2_O_3_ catalyst, while the metals (i.e., Ni) promote hydrogen-transfer reactions. By extension, different metal oxides have been tested for the valorization of bio-oil. Consequently, several studies have investigated the upgrading processes of bio-fuel production by the use of efficient catalysts. The results revealed that Ni-alloy catalysts are more attractive than single Ni catalysts in hydrodeoxigenation and other processes [21,22,23,24,25,26]. Given the literatures analysis mentioned above, the purpose of this study is to focus on the synthesis and use of nanoparticle catalysts based on the nickel oxides (Ni_x_O_x_) without support for viscosity reduction and in-situ hydrogenation heavy oil during the hydrothermal processes. Therefore, the content of this article consists of two parts; the first will be without the use of support for the hydrothermal processes of heavy oil. Therefore, three nickel-based catalysts (Ni_x_O_x_) have been synthesized; they were characterized using different techniques, such as Scanning Electron Microscopy (SEM), Transmission Electron Microscopy (TEM), Atomic Force Microscopy (AFM), and X-Ray Diffraction (XRD) to evaluate their size, morphology, and the metal phases created. The characterization of the properties of the catalysts is very important for the design and manufacture at experimental and industrial scales, as well as for the optimization of the catalytic processes for their possible application at the industrial level. Thus, four experiments (including one (1) without and three (3) with Ni_x_O_x_ nanoparticle catalysts) were carried out in a discontinuous reactor at 300 °C for 24 h of treatment of Ashalcha heavy crude oil from the Republic of Tartarstan (Russia). Characterization of the Ashalcha crude oil was also carried out in order to obtain a baseline of the physical and chemical properties and composition to establish a comparison of the upgrading performance and catalytic activity. Firstly, reaction tests in a reactor of Ashalcha crude oil without catalysts were carried out using only water. Secondly, reaction tests in the reactor were carried out with a catalyst ratio of 2% of the total weight of the heavy crude oil, and then distilled water. Characterization and evaluation of the efficiency and performance of the catalysts were carried out by analyzing the properties of the upgraded oil, including SARA fractions, viscosity, elemental analysis, gas chromatography, GC analysis of saturates, GC-MS measurement of aromatics, and NMR analysis of resins and asphaltenes.

## 2. Experimental Sections

### 2.1. Synthesis of Catalysts

The thermal decomposition synthesis [27,28,29] was carried out in a water solution from [Ni(NH_3_)_6_]Cl_2_ with no surfactants, stabilizers, or organic molecules. A total of three catalysts were synthesized. The first synthesis (denoted as catalyst-1) involved the decomposition of [Ni(NH_3_)_6_]Cl_2_ at 80 °C in the presence of NaOH, with subsequent calcination of the product at 370 °C. The second synthesis (denoted as catalyst-2) involved the decomposition of [Ni(NH_3_)_6_]Cl_2_ at 90 °C without NaOH, with subsequent calcination of the product at 370 °C. The third NiO catalyst (denoted as catalyst-3) was synthesized through a reaction between NiCl_2_ and NaOH at 80 °C, with subsequent calcination of the product at 370 °C.

### 2.2. Characterization of Catalysts

#### 2.2.1. AFM Analysis

**Figure 1:**
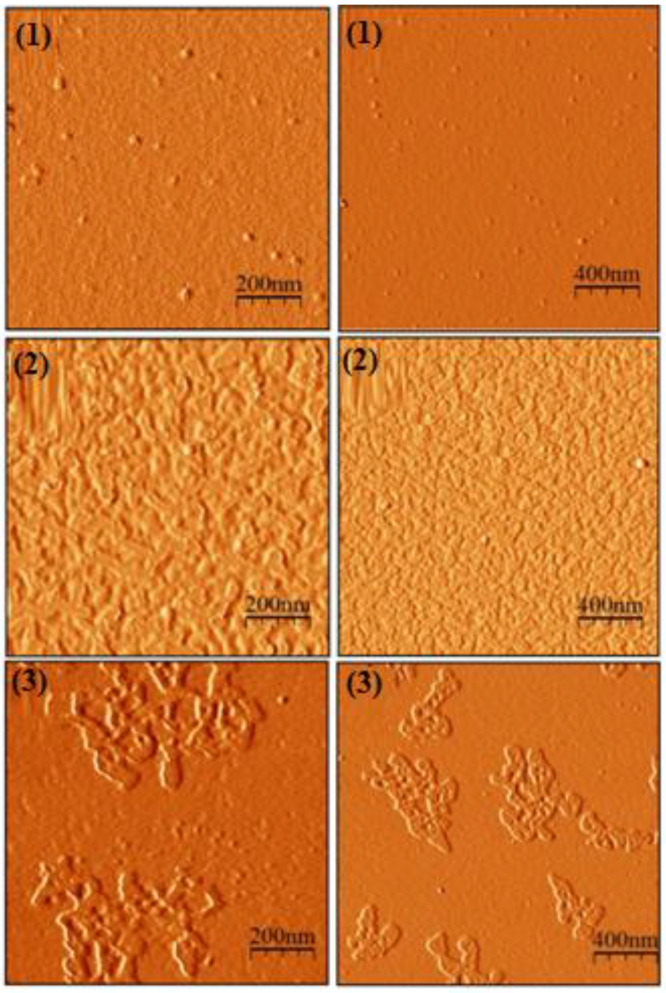
AFM images of catalyst-1 (**1**), catalyst-2 (**2**), and catalyst-3 (**3**) at different magnifications.

**Figure 2:**
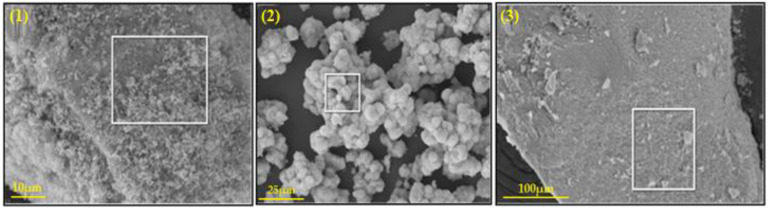
SEM image of catalyst-1 (**1**), catalyst-2 (**2**), and catalyst-3 (**3**). White rectangle depicts the area of EDX data collection.

The results of the AFM analysis of the three catalysts under study are presented in Figure 1. The images obtained by AFM show the change in the morphology and average roughness of each of the catalysts. In general, it was observed that the catalyst that presented less variation in its morphology and roughness is catalyst-1; it was observed that its surface was smooth, with few incrustations or deformations in its area. The opposite happened with catalyst-2, where its entire surface showed a high roughness; however, this characteristic is homogeneous throughout its area. Finally, the NiO catalyst showed high peaks and deformations in some specific areas of its surface; nevertheless, it was not homogeneous, because other sectors with a very low roughness were also observed. AFM analysis was performed in order to characterize the morphology of catalyst-3. For this, catalyst-3 powder was dissolved in deionized water and drop cast on a mica surface. The resulting surfaces were probed by AFM after solvent evaporation in mild conditions. Samples 1 and 3 were found to have platelet-like or flake-like forms, with tracery edges and average dimensions of 60–100 nm and 100–200 nm, respectively. Interestingly, the sample flakes of catalyst-3 were noticeably larger than the samples of catalyst-1. They were distributed separately on the surface and pierced, unlike the plates of the catalyst-1 samples, which were plain and agglomerated, overlapping each other, which seriously decreased the specific surface area. Well-defined single nanoparticles of the catalyst-2 sample, randomly distributed on the surface of the mica wafer, are illustrated on Figure 2b at different magnifications. According to AFM, catalyst-2 grains have a pretty uniform quasi-spherical shape, with dimensions of 8–26 nm and an average diameter of 15 nm.

#### 2.2.2. SEM Analysis

The results of SEM analysis of the three (3) catalysts, namely morphological structure and the chemical composition of the elements, are presented in Figure 2 and Table 1, respectively. It can be observed that catalyst-1 has a higher percentage of Ni in its structure, reaching 79.51 wt.%, while catalyst-3 has only 62.05%. The (Si) might come from the glassware in which the nano-catalysts were synthesized. Additionally, the content of oxygen was determined by recalculation of the elemental analysis results using SEM.

#### 2.2.3. XRD Analysis

**Figure 3:**
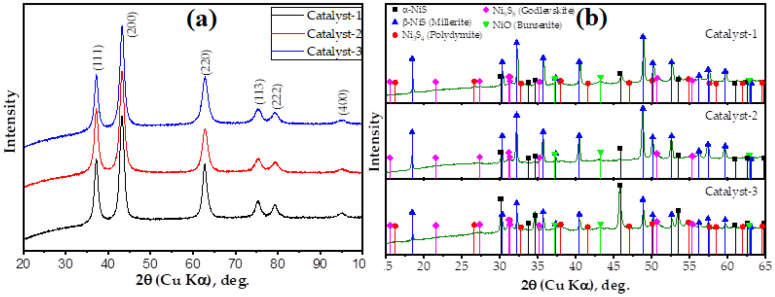
XRD patterns of (**a**) the fresh nickel oxide-based catalysts and (**b**) the solid products obtained after the upgrading of oil using these catalysts. In (**a**), the Miller indexes corresponding to the NiO bunsenite phase are signed, whereas in (**b**), reflexes attributed to the various crystalline phases are labelled by the colored marks.

The X-ray Diffraction (XRD) patterns of the three studied catalysts are shown in Figure 3a, where the black, red, and blue curves correspond to catalyst-1, catalyst-2, and catalyst-3, respectively. The set of reflexes attributed to the NiO bunsenite phase [30] are represented in these patterns. No other crystalline phases were observed. This suggests there is a phase purity of the prepared catalysts. The observed reflexes are broadened, which is probably due to the small sizes of the NiO crystallites. The average crystallite sizes were calculated using Scherrer’s equation considering a shape factor value of 0.9 [31]. The sizes were found to be 7.2 (6) nm, 6.3 (9) nm, and 6.0 (1) nm for catalyst-1, catalyst-2, and catalyst-3, respectively.

#### 2.2.4. XRD of Nanoparticles after Reaction

The solid products obtained after the oil upgrading using the nickel oxide-based catalysts were investigated by the XRD method. The corresponding XRD patterns are shown in Figure 3b. It was found that the nickel sulfide phases of various stoichiometries arose after the oil treatment. The observed reflexes are relatively sharp. This shows that relatively large crystalline nickel sulfide clusters were formed during the oil upgrading process. It should also be noted that the reflexes corresponding to the hexagonal α-NiS phase, which is metastable at room temperature, appear in the XRD patterns. The crystalline nickel sulfide clusters observed in the XRD patterns confirms the reasons for the viscosity reduction reported in the Section 4.2. An example of such an XRD diagram can be found in [32].

#### 2.2.5. TEM Images Analysis

**Figure 4:**
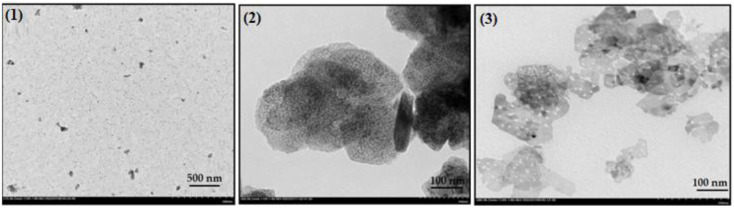
TEM image of catalyst-1 (**1**), catalyst-2 (**2**), and catalyst-3 (**3**).

The results of the TEM analysis of the three catalysts under study are presented in Figure 4. For catalyst-1, a very good resolution is not evident; however, it is possible to appreciate that the nanoparticles are monodisperse spherical, with an average particle size of approximately 40 nm. In the case of catalyst-3, polydisperse particles with diameters ranging from 50 nm to approximately 60 nm are observed, and there is also evidence of a significant grouping of large elements, which would make it possible for the micropore volume to be higher. Catalyst-2 is the one with the highest percentage of Ni in its structure. The images show spherical aggregates with an average particle size of 38 nm. TEM was utilized to support the AFM data. It provides detailed information regarding the morphology of nano objects with high-resolution and detail. In a dried state, catalyst-2 represents weakly aggregated quasi-spherical species scattered on the surface of a copper grid coated with polymeric formvar film. Contrary to that, samples 1 and 3 are flakes with dimensions of 158 nm and 67 nm, respectively. The nanoflakes of sample 1 tend to aggregate and layer on each other, forming piles and stacks, as can be clearly seen in Figure 4. The peculiarity of nickel(II) oxide 3 is in its punctured nature and lack of tendency to form multilayers. This is a prerequisite for the better access of reagents to the active surface area of the catalyst and, hence, better catalytic performance. In this regard, we can conclude that the TEM images are in full agreement with the AFM illustrations.

#### 2.2.6. Adsorption and Desorption Analysis

**Figure 5:**
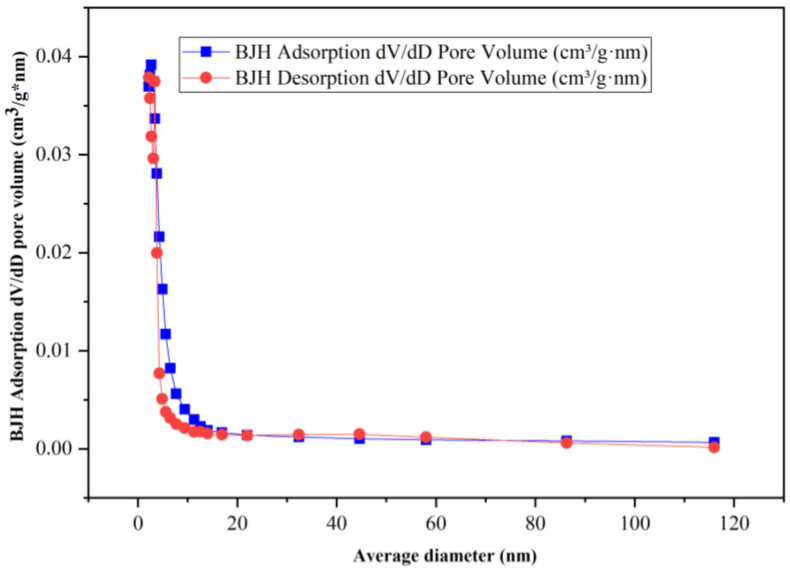
BJH Adsorption and desorption pore volume of catalyst-1.

**Figure 6:**
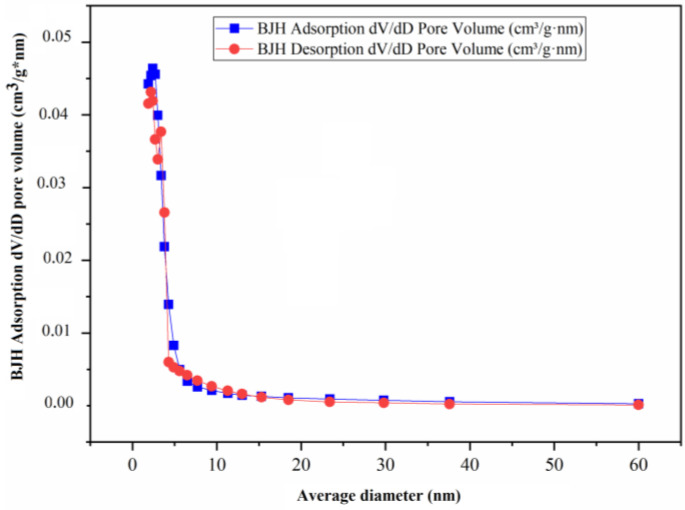
BJH Adsorption and desorption dV/dD pore volume of catalyst-2.

**Figure 7:**
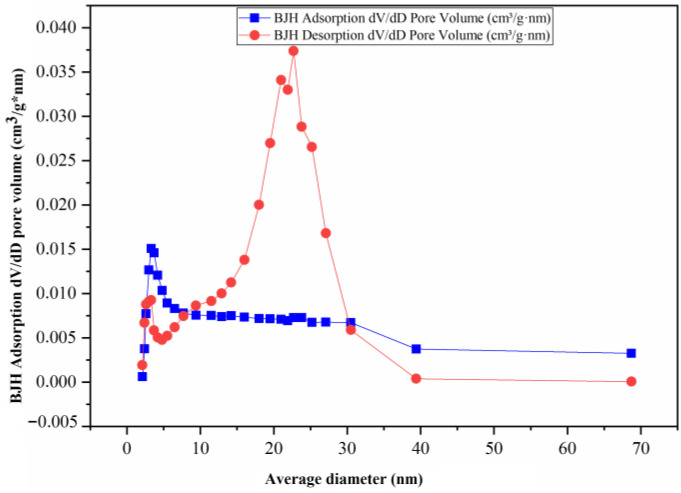
BJH Adsorption and desorption of the pore volume of catalyst-3.

One of the most important characteristics to define in the study of catalysts is that their size, volume, and surface area affect the upgrading of heavy crude oil; therefore, Table 2 summarizes the most important characteristics of the nickel-based catalysts, among which are their micropore volume, total surface area (BET), micropore area, external surface area, and average nanoparticle size. In addition, Figure 5, Figure 6 and Figure 7 show the adsorption and desorption performance of the catalysts (catalyst-1, catalyst-2, catalyst-3), respectively, and Figures S1–S3 from the supporting information show the isothermal behavior for each catalyst. The isotherms are composed of an adsorption process and a desorption process. When the desorption process does not coincide with the adsorption process, hysteresis occurs. These isotherms directly inform the adsorbed volume at a given pressure and allow one to calculate the surface area of the solid, the pore size and its distribution, the heat of adsorption, etc. Five types of isotherms can be distinguished, corresponding to five different classes of solids. The classification is based on the different interactions that the solid may have with the adsorbate and is therefore related to the porosity of the solid. Volumetric methods are generally used to determine adsorption isotherms. For this purpose, a certain known amount of N_2_ is introduced into a vessel containing the adsorbent. The volume of adsorbed gas at equilibrium pressure is the difference between the volume of gas introduced and the volume of adsorbed gas at equilibrium pressure, the adsorption isotherm being constructed point by point by introducing successive charges of gas, allowing sufficient time for equilibrium at each point. Helium is normally used to determine the dead volume. For all the above and according to the results it can be defined that the particle size corresponding to all the catalysts is adjusted to a mesopore because its measurement, taking as reference the pore mouth, is between 2 and 50 nm, approximately. Analyzing the Isotherm linear plot of adsorption and desorption of the NiO catalyst, it can be observed that the curve fits to a type II model, according to Matthias [8], where the adsorption and desorption curves are separated from the highest value, generating a hysteresis with a small area. The hysteresis with a low area can give us an indication that the surface area of the material is not very high, a value that coincides with Table 2, where it is evident that this is the catalyst with the lowest BET area value and is around 106.73 m^2^/g. The opposite occurs for catalyst-1, as represented in Figure 7, where the adsorption and desorption curves coincide in their path (without hysteresis), which corresponds to the gas adsorption on non-porous or macro-porous materials with an unrestricted monolayer-multilayer adsorption up to a high p/p_0_. The observed pore network consists of macropores, which are not filled with pore condensate. The catalyst demonstrating the largest BET area is catalyst-2, and is around 157.96 m^2^/g.

## 3. Methods and Materials

### 3.1. Materials

The heavy crude oil sample was received from Tatneft Oil Company (Kazan, Russia), from a heavy oil deposit from Tatarstan, Russia. The properties and composition of the heavy crude oil are shown in Table 3. The heavy oil has a high sulfur content of approximately 4.21%. The solvents *n*-heptane (99.6%), toluene (98.5%), and isopropyl alcohol (98.8%) were provided by Eco.1 Company (Tathim-product, Kazan, Russia) for SARA analysis, according to ASTM D-4124. The provided chloroform by Eco.1 was also used as a solvent to collect liquid and solid products after the upgrading processes.

### 3.2. Catalytic and Non-Catalytic Hydrothermal Upgrading of Heavy Oil in Reactor

The experiments of the catalytic and non-catalytic upgrading of heavy oil were carried out in a stainless discontinuous reactor in an aquathermolysis condition of 300 ± 1 °C and 67–72 ± 0.3 bars for 24 h of reaction time. For the experiments, 70 g of heavy oil, 30 g of distilled water, and 0.21 g of each catalyst were loaded into the reactor. Molecular nitrogen (N_2_) was used as a carrier gas and medium to remove impurities and provide an initial pressure of 2 bars. Pressure and temperature were recorded automatically. The reactor was cooled down to 100 °C after reaching the planned time and then cooled down to 25 °C by cold water. The evolved gas was analyzed by gas chromatography (Hromatek-Crystal 5000.2 (Yoshkar-ola, Russia)) using GOST 32507-2013 (ASTM D 5134-98 (2008) [33]. Description of samples: Heavy crude oil: feedstock sample; Oil + steam: sample of heavy crude oil + steam without catalysts; Oil+ catalyst-1: sample of heavy crude oil + steam + catalysts 1; Oil + catalyst-2: sample of heavy crude oil + steam + catalysts-2; Oil + catalyst-3: sample of heavy crude oil + steam + catalysts-3.

### 3.3. Analytical Methods

#### 3.3.1. Viscosity Measurement

The obtained products after heavy crude oil upgrading using aquathermolysis conditions were evaluated using various methods. A Brookfield DV-II + Pro (Fungilab) viscometer (Barcelona, Spain) and SVM 3000 t (Anton Paar, Houston, TX, USA) viscometer were used to determine the viscosity and API gravity of liquid products, respectively. For all analyses, it was necessary to use a TL6 spindle at 25 °C and a sample volume of 9.0 mL [34].

#### 3.3.2. Elemental Analysis

Analysis of elemental chemical composition (C, H, S, N, and O&Me) of heavy crude oil and of upgraded oil was carried out using analyzer PerkinElmer 2400 Series II [35].

#### 3.3.3. GC Measurement of Saturated Fractions

In this study, a Gas chromatograph Agilent 7890B (Santa Clara, CA, USA) was used to determine the molecular distribution of *n*-alkanes in the heavy crude oil and in the upgraded oil. A Gas chromatograph Agilent 7890B equipped with a flame ionization detector was used with a capillary column type HP-5, with a length of 30 m and a diameter of 0.32 mm. The carrier gas (nitrogen) flow rate was 1.5 mL/min. The temperature of the injector was 310 °C. The column temperature was raised at a rate of 10 °C/min from 100 to 150 °C, then at 3 °C/min from 150 to 325 °C, followed by an isotherm at 325 °C until the end of the study. The classification of the compounds was then ensured by the “Agilent ChemStation”, GC Measurement of Saturated Fraction [35].

#### 3.3.4. GC−MS Measurement of Aromatic Fraction

The fractions of aromatics extracted from the heavy crude oil and upgraded oil were analyzed using a Gas Chromatography Mass Spectrometer system (GC-MS), which included the gas chromatograph “Chromatec-Crystal 5000” with an ISQ mass selective detector (Yoshkar-ola, Russia). Xcalibur software was used to process the results. The chromatograph was equipped with a capillary column type CR-5 ms of 30 m length and 0.25 mm diameter. The rate at which the carrier gas (helium) flow was performed was 1 mL/min. The injector temperature was 310 °C. The thermostat temperature program was as follows: temperature increase from 100 to 150 °C at a rate of 3 °C/min and from 150 to 300 °C at a rate of 12 °C/min, followed by its isotherm until the end of the analysis. The electron energy of the mass detector was 70 eV; the ion source temperature was 250 °C. Compounds were identified through the electronic library of the NIST spectra database and based on data from literature sources [35].

#### 3.3.5. NMR Spectroscopy Measurement

The asphaltenes and resins obtained from heavy crude oil and upgraded oil were analyzed with a Bruker AVANCE-III-HD-700 ^13^C NMR spectrometer, Lethbridge, Alberta, Canada. Field blocking and stagnation were realized using the deuterium D_2_O signal in a glass capillary placed in a 5 mm NMR tube. The resin’s fractions were diluted in deuterochloroform to obtain a dilute solution. The 1H NMR spectra were registered at 25 °C (zg30 pulse program), the acquisition time was 4.7 s, the pre-scan delay was 6.5 μs, the time between scans was 2 s, the spectrum width was 12.0 ppm (6000 Hz), and 400 scans were collected. The ^13^C NMR spectra were measured using 90 °C pulses with reverse gated broadband proton decoupling. The relaxation time between pulses was 9 s (and the acquisition time was 3.5 s), the spectrum width was set to 220.0 ppm, and the total scans was 3200. A digital exponential filter with the line broadening parameter of 10 Hz (lb) was applied to process the ^13^C NMR spectra before Fourier transformation. Measurements were performed at a temperature of 25 °C. All ^13^C NMR spectra were integrated after baseline correction, and an average of the on-screen integration values was taken for each calculation [34].

## 4. Results and Discussion

### 4.1. Analysis of the Evolved Gases

The results of composition and yield of the released gaseous products after the catalytic and non-catalytic hydrothermal upgrading processes of heavy oil are illustrated in Table 4. It can be seen that the released gases are mainly composed of C_1_–C_4_, H_2_, H_2_S, CO, and CO_2_. It should be noted that the catalytic and non-catalytic hydrothermal upgrading processes of heavy oil are accompanied by intensive cracking, including sulfur compound C-S cleavage through hydrodesulfurization reaction, from which a maximal yield of H_2_S of 1.2913% was observed in the sample in the absence of catalysts (without catalyst). Meanwhile, the use of nanoparticles of nickel oxide catalysts in the upgrading processes of heavy oil reveals, on the one hand, a good selectivity for molecular hydrogen production (H_2_) by dehydrogenation, compared with that of hydrodesulfurization for H_2_S production. The slightly increased hydrogen yield could be related to the presence of Ni particles, which can cause a steam reforming reaction/water gas shift reaction; the increase in value (H_2_/CO) is proof of this [35,36]. The selectivity in the total yield of hydrocarbon gases (∑C_1_–C_4_) trend towards maximal values in the samples without catalyst of 2.5798%, and of 2.9961% in the sample with catalyst-1. The reasons for the increase in the yields of ∑C_1_–C_4_ and molecular hydrogen (H_2_) are related to the cleavage of C-C and dehydrogenation of the C-H bonds [37,38], and to the low hydrogenation rate of the liquid products (without catalyst and with catalyst-1). This is in agreement with the results of the elemental analysis (Table 5), which revealed that the contents of the hydrogen atomic (H) have a lower value. Regarding the release of gases (CO and CO_2_), overall, the yield of CO_2_ increases more in the catalyst-1 and catalyst-2 samples, which is due to a rise in part from the methane reforming reactions, with steam producing the syngas product; the reaction is described in [34,39]. Another important reaction to generate CO_2_ is the decarboxylation of carboxylic acids from heavy oil. Both reactions can lead to the increase of CO_2_ [35,40].

### 4.2. Viscosity of Heavy and Upgraded Oil

**Figure 8:**
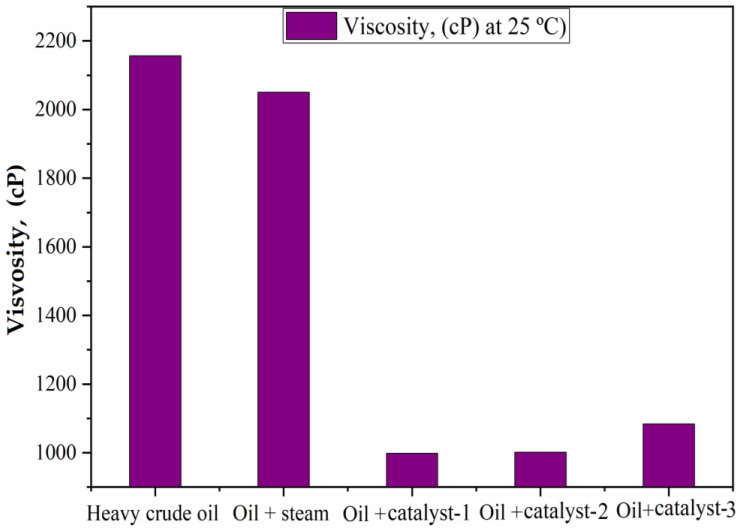
Viscosity of heavy oil and upgraded oil samples.

Figure 8 shows the results of the viscosity analysis of the heavy crude oil and the upgraded oil samples. After the hydrothermal and catalytic upgrading processes, the viscosity of the heavy crude oil was significantly reduced, from 2157 mPa·s to 2050.6 mPa·s, for the sample of the upgraded oil without catalyst, and then to a lower value of 998.6 mPa·s for the sample with catalyst-2. It is accepted by many authors [19,37] that the significant reduction in the viscosity of heavy oil during hydrothermal upgrading processes is mainly due to the cleavage of C-heteroatom bonds (such as C−S and C−N) as well as some C−C bonds contained in the large molecules (resins and asphaltenes). Consequently, in this work, in the elemental analysis (Table 5) it is revealed that the heteroatom components (S,N) decreased after hydrothermal upgrading; this directly coincides with the viscosity decrease of heavy crude oil. Secondly the reason for the decrease of viscosity can be explained by the redistribution of hydrogen over carbons, and this can observed by the increase of H/C; see Table 4. Moreover, it is widely believed that even a small amount of bond cleavage (such as the C−S bond) may lead to a large reduction in viscosity [18]. Thirdly, the reduction in viscosity of heavy oil can be supported by the change in SARA fractions, i.e., a significant increase in saturates and an obvious decrease in aromatics and resins.

### 4.3. Analysis of Chemical Elements (C, H, N, S, and O&Me)

Table 5 illustrates the results of the composition of chemical elements of the catalytic and non-catalytic upgraded oil. The results show a decrease in the content of redistributed carbon after the hydrothermal upgrading process, which reaches a minimum value in the sample with catalyst-3. It has been observed that, under hydrothermal conditions and catalytic addition, the carbon content tends to decrease after the hydrothermal upgrading of heavy oil, and this can be related to the cracking of the C-C bonds to form low molecular weight gas or to a slight formation of coke on the catalyst area. Therefore, given the use of high adsorption performance catalysts based on nickel according to the BET results mentioned above, the atomic hydrogen content increased in all the upgraded samples, except for the sample with catalyst-1, which decreased. Similarly, the heteroatom and metal (N, S, O&Me) content also decreased due to hydrodesulfurization, denitrogenation, and hydrogenolysis reactions. The H/C ratio determines the hydrogenation degree of the carbon during the upgrading process, and in this study it is increased relative to that of the heavy crude oil sample. However, it has been found that the H/C ratio is increased in all samples and reaches a maximum value of 1.77 with catalyst-2, from 1.48 for heavy crude oil. This increase may explain or strengthen the reasons why the viscosity of the upgraded oil samples decreases, as well as the increase in the percentage of light hydrocarbon fractions (saturates) according to the SARA analysis. Therefore, we need to conclude that the best catalyst from the three catalysts for the in-situ hydrogenation (hydrogen distribution over carbon in liquid phase) upgrading process is catalyst-2.

### 4.4. SARA Analysis

**Figure 9:**
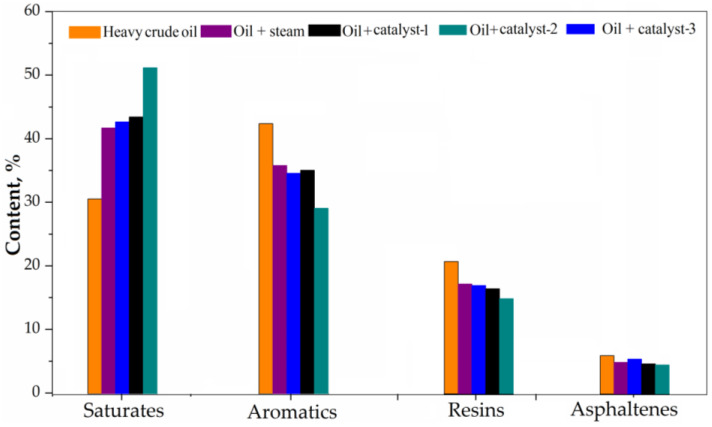
SARA fractions of heavy crude oil and upgraded oil samples.

**Figure 10:**
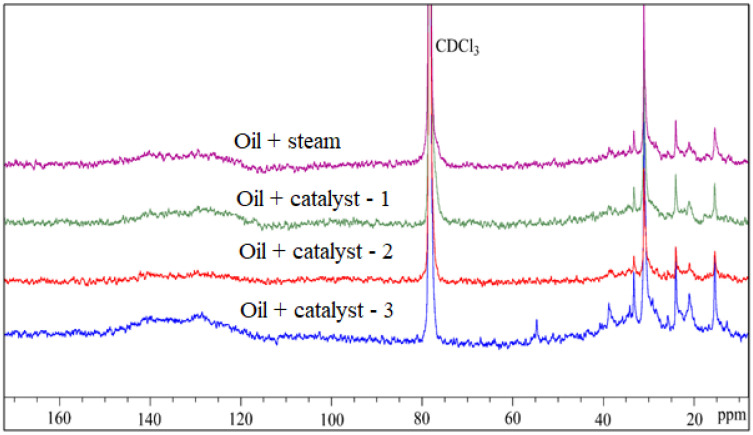
^13^C NMR spectra of resin fractions of original and upgraded oil.

**Figure 11:**
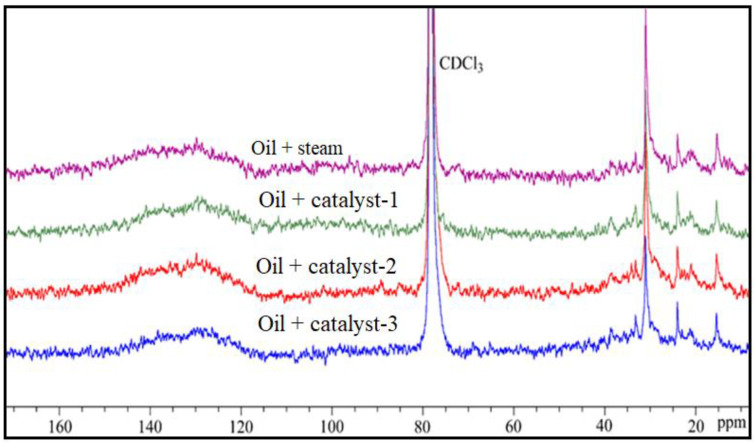
^13^C NMR spectra of asphaltene fractions of original and upgraded oil.

To have an idea of the efficiency of the hydrothermal upgrading process of heavy crude oil and the distribution fractional components of light and heavy molecules, analysis of SARA fractions of heavy crude oil and upgraded oil samples were carried out, and the results are shown in Figure 9. After the upgrading processes, the high molecular weight fractions decrease with the increase in light fractions, also with heteroatoms and metals removed. Therefore, it has been observed that after the catalytic and non-catalytic upgrading processes, the content of saturates has significantly increased from 30.65%—heavy crude oil, to 41.83%—without catalyst, 42.76%—with catalyst-3, and 43.56%—with catalyst-1, and reaches a maximum of 51.30%—with catalyst-2. The increase in saturate fractions can be explained by a series of reactions produced during upgrading, including isomerization, alkylation of n-alkanes, dealkylation of alkyl aromatic structure, and the hydrogenation of aromatics, which can be verified by the results of ^13^C NMR described (see Section 4.7 and Figure 10 and Figure 11). Given these observations, the content of aromatic fractions decreased after upgrading due to the dealkylation by cracking of the benzene ring; the results of the ^13^C NMR prove there is an increase in primary (-CH_3_) groups in the resins fractions and a decrease of secondary and quaternary (-CH_2_- and =C=) groups in the asphaltenes fractions. Additionally, there is a hydrogenation of aromatics that convert aromatics into saturates; on the other hand, these can also be resulted from their condensation reactions to form asphaltenes [12,34,35]. In the case of resins, their content was significantly reduced as a result of the upgrading, as demonstrated by the ^13^C NMR. The cleavage of C-heteroatom bonds, as well as some C−C bonds in the large molecules of resins, could be the most important reaction to convert resins to smaller molecules (saturated gases, smaller resin structure, etc.), which is also one of the principal reasons why the viscosity was significantly reduced, as mentioned previously. Another reaction pathway to consume resins is the same as for aromatics, that is, their condensation reactions. The asphaltene content also decreased, and the reason for this could also be due to the fact that the thermal cracking of heavy oil is a very complex process involving the simultaneous occurrence of several parallel and consecutive reactions, such as cleavage, polymerization and condensation, dehydrogenation, etc. [2].

### 4.5. GC Measurement of Saturated Fraction Upgrading Oil

Table 6 and Figures S4–S8 from the supporting information illustrate the results of the carbon number distribution of n-alkanes in saturate fractions by the chromatograms of GC measurement. In this study, to simplify the interpretation of the results, the saturates were grouped into four fractions, which are C_8_−C_15_, C_16_−C_25_, C_26_−C_35_, and C_36_−C_38_. In general, the content of the C_8_–C_15_ and C_16_–C_25_ alkanes, considered as lighter fractions, increases significantly, and that of C_26_–C_35_ and C_36_–C_38_ decreases after the upgrading processes compared to the initial heavy oil, as shown in Table 5. The increase in C_8_–C_15_ and C_16_–C_25_ can mainly be attributed to the rupture of long-chain alkanes and the dealkylation of aromatics in resins and asphaltenes. In particular, for the catalyst-3 sample, the highest content of C_8_–C_15_ and C_16_–C_25_ and the lowest content of C_26_–C_35_ and C_36_–C_38_ were observed. Moreover, comparing this result with the data of SARA fractions, it can be seen that, after upgrading, saturates also show the highest content and asphaltenes show the lowest content. Therefore, it can be assumed that the increase of the saturates and the content of the light fractions C_8_–C_15_ and C_16_–C_25_ is an overall result of the thermal and catalytic cracking reactions as a whole. For example, for the case of asphaltenes, it can first be explained by the fact that it can be destroyed by side chain scission and ring opening reactions; secondly, it can be generated by condensation and polymerization reactions; and thirdly, it can be transformed into coke by the dehydrogenation of cycloalkanes, in addition to condensation and rearrangement steps. However, for our test, no coke was obtained.

### 4.6. GC−MS Measurement of Aromatic Fractions

The GC−MS results of aromatics analysis are presented in Table 7. In this study, the aromatics fractions are divided into mono-, di-, and polyaromatic (MDPA), as well as unspecified types according Djimasbe et al. [34]. Generally, the mono-aromatic fractions decreased after the hydrothermal upgrading processes of heavy oil, from 61.45% to practically 0%, except for the sample with catalyst-3, in which a low amount of monoaromatic fractions of approximately 8.20% was observed. Meanwhile, in the diaromatic fractions, the content increased in all upgraded samples, and a maximum of 66.39% was observed in the sample without catalyst. This increase in diaromatic fractions by the consumption of the monoaromatic fractions could be the result of the dehydrogenation of cycloalkane (decalin and tetraline) structure molecules, the cyclization of long-chain aliphatic radicals, and the cracking of asphaltenes fractions; this has been verified by the results of the SARA analysis. According to the thermal cracking behavior of aromatics, it is also reasonable to assume that the change in mono-, di-, and poly-aromatic content must also be affected by the scission of large molecules (resins and asphaltenes).

### 4.7. NMR Analysis of Resins and Asphaltenes of Upgraded Oil

The results of the ^13^C NMR spectra of resins and asphaltenes in the initial and upgraded oil are shown in Figure 10 and Figure 11, and the results are summarized in Table 8 and Table 9, respectively. The values of Cp, Csq, Ct, and Car were obtained by integrating the corresponding regions in the ^13^C NMR spectra. The aromaticity factor (FCA) and aromatic (Car carbons) decreased from 0.42 to 0.08, and from 41.98 to 20.22, respectively. In addition, the primary group (-CH_3_, Cp) and the tertiary group (C_t_) increased, while the secondary and quaternary group (Csq) decreased. The ^13^C NMR data of the asphaltenes (Table 8 and Figure 11) show similar results to those of the resins. All these indications suggest that a rearrangement of the secondary functional groups (-CH_2_-) occurred, resulting in a decrease in the number of CH_2_ in the side chains of the aromatics, and simultaneously isomerization, alkylation, hydrogenation, and ring opening reactions occurred, leading to an increase in Cp and C_t_. These reactions are also the main pathways that convert resins and asphaltenes into saturates, which confirms the previous conclusion on the change in SARA fractions. Furthermore, these results partly demonstrate the results of the GC analysis for saturates that C_26_–C_35_ alkanes crack into C_16_–C_25_ and C_8_–C_15_ alkanes by scission of the C–C bond, as well as the formation of cycloalkanes by the hydrogenation of aromatics. Obviously, the use of catalysts promoted these mentioned reactions for the conversion of resins and asphaltenes into lighter fractions, such as saturates, while inhibiting the condensation, polymerization, and recombination processes. Therefore, to conclude, the ^13^C NMR results confirm that the introduction of a catalyst into the heavy oil upgrading process caused predominant isomerization, alkylation, hydrogenation, and ring opening reactions, which improved the quality of the upgraded oil.

## 5. Conclusions

The synthesis of nanoparticle catalysts based on nickel (NixOx) has been successfully carried out in this work, and their testing in the hydrothermal upgrading processes of heavy oil for viscosity reduction has also been carried out. The results revealed allow us to draw the following conclusions: According to SEM and TEM results, all of the obtained catalysts have nano-scale sizes of particles, ranging from 37 to 56 nm, and the total surface area BET ranges from 106 to 157 m^2^/g. It can be seen that the largest BET area was observed for catalyst-2, with approximately 157.96 m^2^/g. Given the high catalytic activity and the largest BET area of the catalyst, approximately 9.64% mol were observed after the upgrading process of heavy oil, namely for catalyst-2. The performance of the upgrading process of heavy oil using nickel-based catalysts was proven by a significant viscosity reduction, from 2157 cP to lower than 1000 cP, as well as a not negligible increase of the saturate fraction, with a reduction of aromatics, resins, and asphaltenes. The upgraded oil contained saturate fractions ranging from 30.65 to a maximum of 51.30 wt.%, with a large reduction of resins and asphaltenes from 20.82 and 6.03 wt.% to 14.95 and 4.56 wt.%, respectively. In particular, it has been observed that the upgraded oil samples using nickel-based catalysts were significantly characterized by a high redistribution of hydrogen in carbon, and consequently the maximum value of H/C (1.77) was observed in the catalyst-2 sample. Redistribution of n-alkanes fraction C_8_–C_15_ (light fractions) content was significantly increased to 22.52 from 17.05%, and C_16_–C_25_ increased to 49.69 from 42.51%, while high-molecular weight alkanes (C_36_–C_38_) content was decreased to 4.08 from 14.34%. The heteroatom compounds (S, N) were also significantly decreased in the upgraded oil samples. The NMR ^13^C results of resin and asphaltene fractions revealed that the decomposition of high molecular weight compounds occurred through the cracking of C-C bonds, isomerization, and dealkylation of the alkyl aromatics structure. According to the results obtained, the sample that showed the best performance was the catalyst-3 sample, corresponding to the upgrading oil with catalyst-2. Given all the obtained results, the use of nickel oxide-based catalysts has direct and beneficial effect on the upgrading process of heavy crude oil, due to the high catalytic activity of the catalysts, and all these factors gives its wide application great potential to catalyze the aquathermolysis reaction in the steam injection process during in-situ upgrading for the recovery of heavy oil.

## Figures and Tables

**Table 1:** Chemical composition of nickel catalyst using EDX.

Elements, %	Catalyst-1	Catalyst-2	Catalyst-3
O	20.06	25.95	37.01
Si	0.43	0.27	0.94
Ni	79.51	73.79	62.05

**Table 2:** Parametric characteristics of nickel oxide catalysts samples.

Parameter	Catalyst-1	Catalyst-2	Catalyst-3
Micropore volume: cm^3^/g	−0.0122	−0.016121	0.002238
Total Surface area (BET): m^2^/g	152.80	157.96	106.73
Micropore area: m^2^/g	-	-	7.87
External surface area: m^2^/g	172.18	187.11	98.85
Average Nanoparticle Size (nm)	39.27	37.98	56.22

**Table 3:** Properties, chemical composition, and SARA fractions of heavy crude oil.

API Gravity (°)	Viscosity (mPa·s)	SARA Fractions (wt.%)				Organic Elemental Content (wt.%)				
		Saturates	Aromatics	Resins	Asphaltenes	C	H	S	N	O&M
14.1 ^b^	2157 ^a^	28.8	44.3	21.0	5.9	82.09	10.12	0.40	4.28	3.11

^a^ Viscosity of heavy crude oil was measured at 25 °C and 1 atmospheric pressure using a rotational viscosimeter (Brookfield DV-II Pro, Barcelona, Spain). ^b^ SARA fractions of heavy crude oil were determined according to ASTM D-4124.

**Table 4:** Results of released gases analysis of the hydrothermal upgrading of heavy oil.

Component	Concentration (Mol.%)			
	Oil + Steam	Oil + Catalyst-1	Oil + Catalyst-2	Oil + Catalyst-3
Hydrogen (H_2_)	2.5804	8.6437	9.6471	3.4101
CO_2_	0.6893	1.0890	0.6995	0.3885
Nitrogen (N_2_)	92.710	86.574	86.637	94.372
ΣC_1_–C_4_	2.5798	2.9961	2.0643	1.2421
H_2_S	1.2913	0.5406	0.8753	0.5234
CO	0.1488	0.1569	0.0769	0.0641
H_2_/CO	17.341	55.090	125.449	53.199

**Table 5:** Elemental Analysis of heavy crude oil and upgraded oil samples.

Sample	C, %	H, %	N, %	S, %	(O&Me) *	H/C
Heavy crude oil	82.09	10.12	0.40	4.28	3.11	1.48
Oil + steam	82.46	10.34	0.38	4.24	2.67	1.50
Oil + catalyst-1	81.61	11.28	0.39	3.98	2.74	1.65
Oil + catalyst-2	81.56	12.07	0.37	3.32	2.98	1.77
Oil + catalyst-3	81.75	10.76	0.38	4.13	2.98	1.58

* is determined by difference.

**Table 6:** Distribution of n-alkanes in saturate fraction of crude and upgraded oil.

n-Alkanes	Contents of n-Alkanes, %				
	Heavy Crude Oil	Oil + Steam	Oil + Catalyst-1	Oil + Catalyst-2	Oil + Catalyst-3
**ΣC_8_–C_15_**	17.05	15.33	14.74	13.24	22.52
**ΣC_16_–C_25_**	42.51	52.17	51.11	53.08	49.69
**ΣC_26_–C_35_**	26.10	27.32	28.20	27.68	23.71
**ΣC_36_–C_38_**	14.34	5.18	5.94	6.00	4.08

**Table 7:** GC−MS results of aromatic fractions of crude and upgraded oil.

Aromatic Fractions	Heavy Crude Oil	Oil + Steam	Oil + Catalyst-1	Oil + Catalyst-2	Oil + Catalyst-3
Alkanes, %	0.00	10.20	1.72	1.21	12.63
Mono, %	61.45	0.97	0.00	0.00	8.20
Di, %	21.90	66.39	43.97	58.49	32.66
Poly, %	16.30	19.64	52.47	40.31	34.51
Others, %	0.35	2.80	1.83	0.00	12.01

**Table 8:** Molar Fraction (%) of carbon groups of resins before and after the upgrading process.

Group Type	Molar Fraction (mol%)				
	Heavy Crude Oil	Oil + Steam	Oil + Catalyst-1	Oil + Catalyst-2	Oil + Catalyst-3
C_p_	10.40	14.00	11.50	13.70	14.10
C_sq_	38.80	38.70	37.00	36.80	36.40
C_t_	8.40	21.65	31.80	16.98	29.20
C_ar_	41.98	25.40	20.23	32.20	20.22
F_CA_	0.42	0.25	0.27	0.32	0.08

**Table 9:** Molar Fraction (%) of carbon groups of asphaltenes before and after the upgrading process.

Group Type	Molar Fraction (mol%)				
	Heavy Crude Oil	Oil + Steam	Oil + Catalyst-1	Oil + Catalyst-2	Oil + Catalyst-3
C_p_	15.10	14.20	22.10	23.50	22.30
C_sq_	36.00	16.62	19.30	25.10	24.27
C_t_	8.50	41.00	32.90	31.40	30.40
C_ar_	40.00	27.90	25.39	19.70	22.80
F_CA_	0.40	0.28	0.31	0.30	0.23

## Data Availability

Not applicable.

## Supplementary Materials

The following supporting information can be downloaded at: https://www.mdpi.com/article/10.3390/nano13081351/s1, Figure S1: Isotherm linear plot of adsorption and desorption of catalyst-1; Figure S2: Isotherm linear plot of adsorption and desorption of catalyst-2; Figure S3: Isotherm of adsorption and desorption of catalyst-3; Figure S4: Chromatogram of saturate fractions of heavy crude oil; Figure S5: Chromatogram of saturate fractions of upgraded oil +steam; Figure S6: Chromatogram of saturate fractions of upgraded oil + catalyst-1; Figure S7: Chromatogram of saturate fractions of upgraded oil + catalyst-2; Figure S8: Chromatogram of saturate fractions of upgraded oil + catalyst-3.

## Abbreviations

API, American Petroleum Institute; AFM, Atomic Force Microscopy; Sub-CW, Sub-critical conditions; SARA, Saturates, Aromatics, Resins and Asphaltenes; ASTM, American Society for Testing and Materials; TEM, Transmission Electron Microscopy; SEM, Scanning Electron Microscopy; XRD, X-Ray Diffraction; NMR, Nuclear Magnetic Resonance; GC, Gas Chromatography; GC MS, Gas Chromatography-Mass Spectrometer system; FID, Flame Ionization Detector; TCD, Thermal Conductivity detectors; Cp, Primary (methyl groups−CH_3_); Csq, Secondary (methylene −CH_2_− quaternary; Ct, Tertiary (CH groups); Cal, Total aliphatic carbon content; FCA, The aromaticity factor; MDPA—Mono-, di-, and poly-aromatics.
