# Supplementary material for: Use of Nickel Oxide Catalysts (Bunsenites) for In-Situ Hydrothermal Upgrading Process of Heavy Oil
Source: Nanomaterials (Basel). 2023 Apr 12;13(8):1351. doi: 10.3390/nano13081351 (PMC10143493; doi:10.3390/nano13081351)
Supplement: Supplementary file 1 [file nanomaterials-13-01351-s001.zip › nanomaterials-2282144-supplementary.pdf]

Supporting Information

Article

Use of Nickel Oxide Catalysts (Bunsenites) for In-Situ Hydrothermal Upgrading Process of Heavy Oil

Jiménez Padilla Pedro Alonso [1], Richard Djimasbe [1,*], Rustem Zairov [2,3], Chengdong Yuan [1], Ameen A. Al-Muntaser [1], Alexey Stepanov [3], Guliya Nizameeva [4], Alexey Dovzhenko [2,3], Muneer A. Suwaid [1], Mikhail A. Varfolomeev [1,2,*] and Almaz L. Zinnatullin [5]

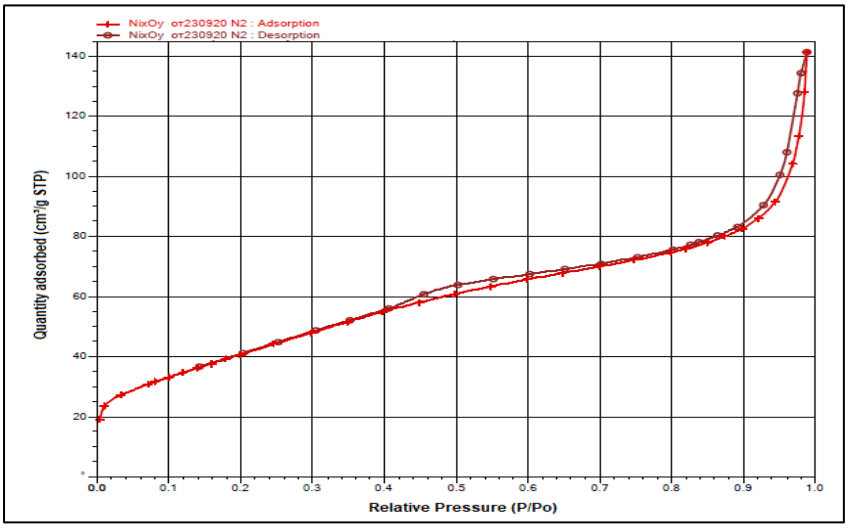

Figure S1. Isotherm linear plot of adsorption and desorption of catalyst-1.

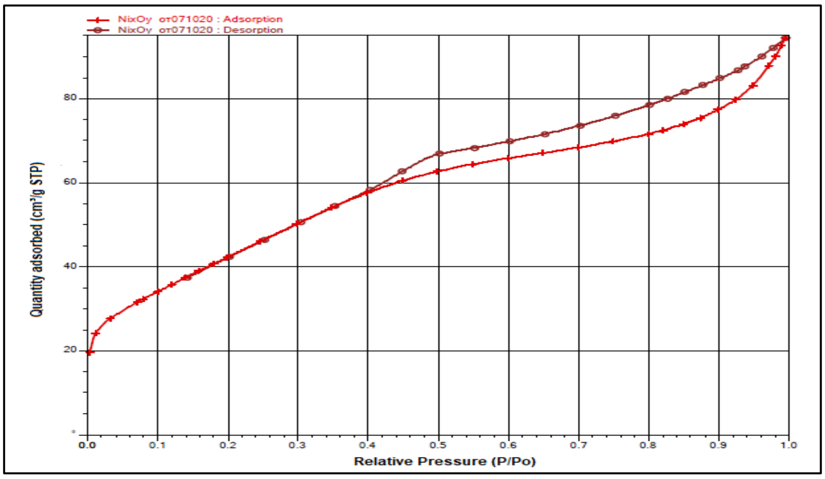

Figure S2. Isotherm linear plot of adsorption and desorption of catalyst-2.

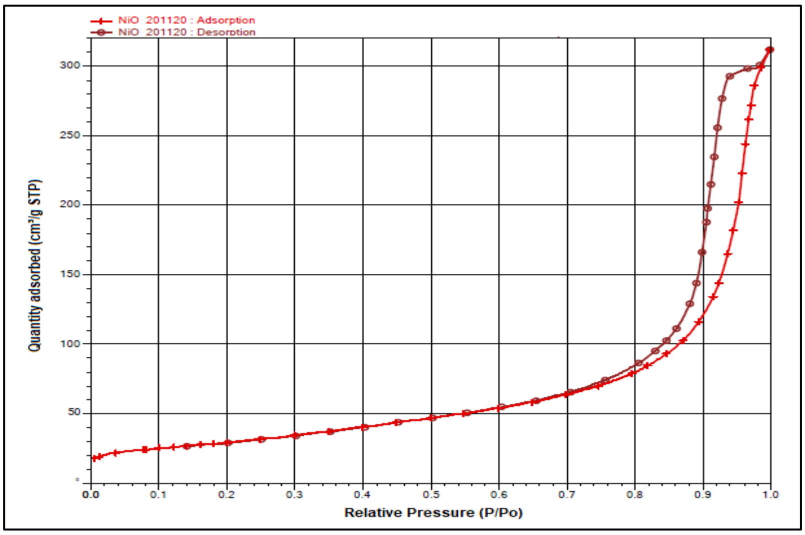

Figure S3. Isotherm of adsorption and desorption of catalyst-3.

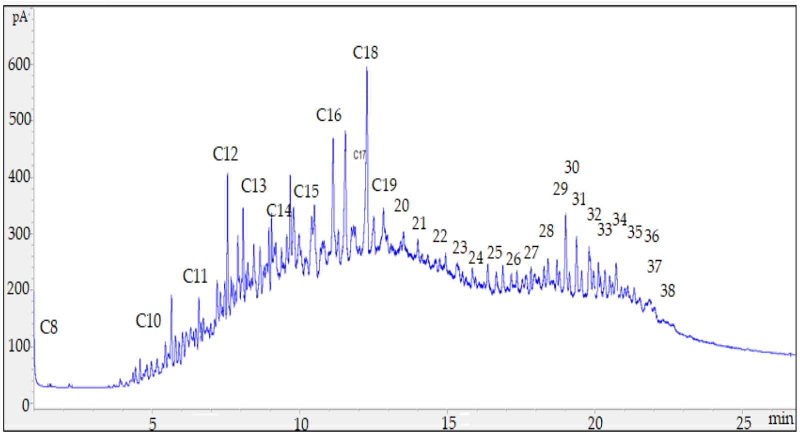

Figure S4. Chromatogram of saturate fractions of heavy crude oil.

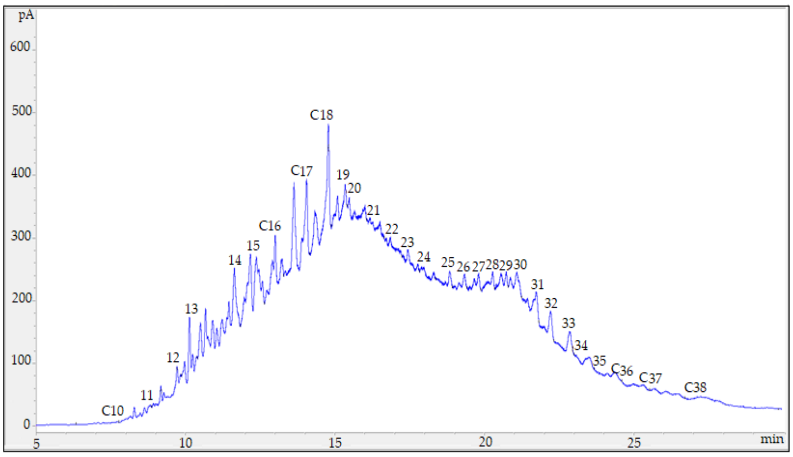

Figure S5. Chromatogram of saturate fractions of upgraded oil +steam.

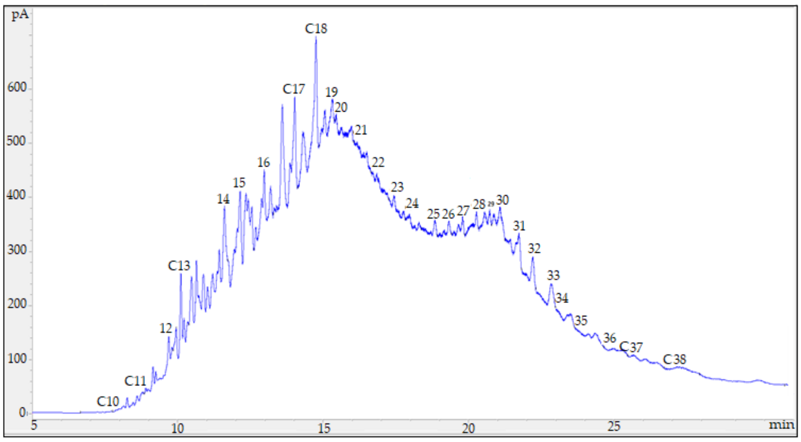

Figure S6. Chromatogram of saturate fractions of upgraded oil + catalyst-1.

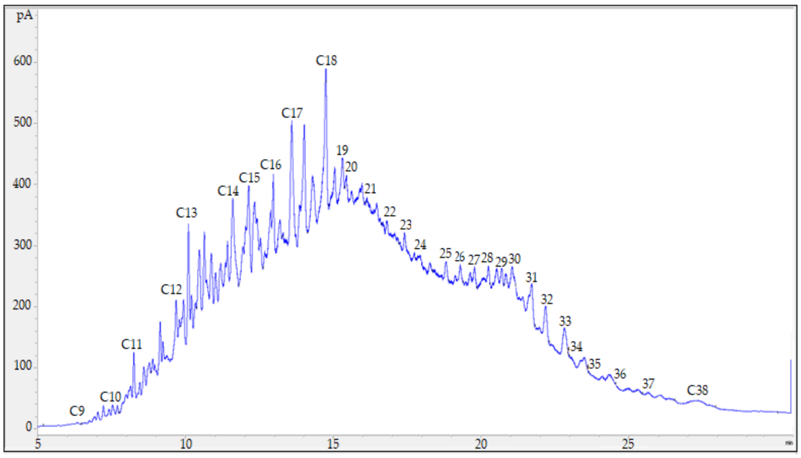

Figure S7. Chromatogram of saturate fractions of upgraded oil + catalyst-2.

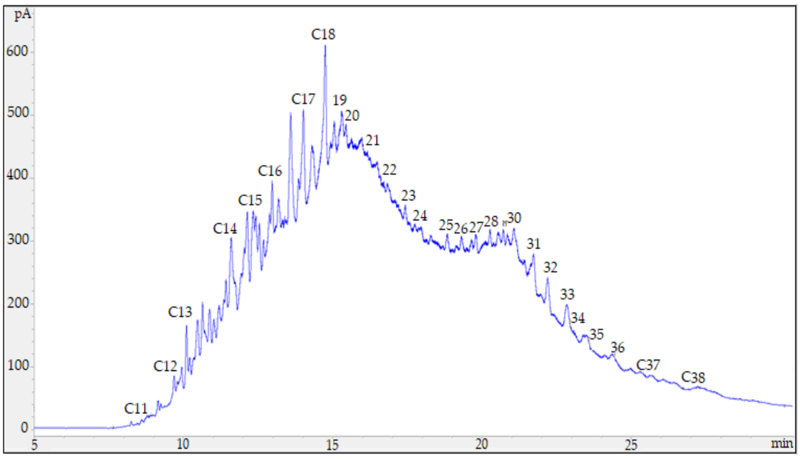

Figure S8. Chromatogram of saturate fractions of upgraded oil + catalyst-3.
